# Use of General Practitioner Services Among Workers with Work-Related Low Back Pain: A Systematic Review

**DOI:** 10.1007/s10926-024-10187-x

**Published:** 2024-04-23

**Authors:** Preeti Maharjan, Asmare Gelaw, Daniel Griffiths, Danielle Mazza, Alex Collie

**Affiliations:** 1 Healthy Working Lives Research Group, School of Public Health and Preventive Medicine, Monash University, Melbourne, Australia; 2 Department of General Practice, School of Public Health and Preventive Medicine, Monash University, Melbourne, Australia; 3 Centre for Health Policy, Melbourne School of Population and Global Health, University of Melbourne, Melbourne, Australia

**Keywords:** Work-related low back pain, Primary care, General Practitioners, Work-related injury, Musculoskeletal disorders

## Abstract

**Purpose:**

Work-related low back pain (WRLBP) is a highly prevalent health problem worldwide leading to work disability and increased healthcare utilisation. General practitioners (GPs) play an important role in the management of WRLBP. Despite this, understanding of GP service use for WRLBP is limited. This systematic review aimed to determine the prevalence, patterns and determinants of GP service use for WRLBP.

**Methods:**

MEDLINE, Embase via Ovid, Scopus and Web of Science were searched for relevant peer-reviewed articles published in English without any restriction on time of publications. Low back pain (LBP) was considered work-related if the study included workers’ compensation claim data analysis, participants with accepted workers’ compensation claims or reported a connection with work and LBP. The eligibility criteria for GP service use are met if there is any reported consultation with family practitioner, medical doctor or General Practitioner. Two reviewers screened articles and extracted data independently. Narrative synthesis was conducted.

**Results:**

Seven eligible studies reported prevalence of GP service use among workers with WRLBP ranging from 11% to 99.3%. Only studies from Australia, Canada and the United States met the eligibility criteria. The prevalence of GP service use was higher in Australia (70%) and Canada (99.3%) compared to the United States (25.3% to 39%). The mean (standard deviation) number of GP visits ranged from 2.6 (1.6) to 9.6 (12.4) over a two-year time interval post-WRLBP onset. Determinants of higher GP service use included prior history of low back pain, more severe injury, prior GP visits and younger age.

**Conclusion:**

Only seven studies met the eligibility indicating a relative lack of evidence, despite the acknowledged important role that GPs play in the care of workers with low back pain. More research is needed to understand the prevalence, patterns and determinants to support effective service delivery and policy development.

**Supplementary Information:**

The online version contains supplementary material available at 10.1007/s10926-024-10187-x.

## Introduction

Work-related low back pain (WRLBP) is a major contributor to years lived with disability affecting millions of workers globally [1, 2]. The Global Burden of Disease 2020 study reported that WRLBP was responsible for a total of 194 years lived with disability per 100,000 population, with its prevalence projected to increase by 36.4% between 2020 and 2050 [2]. Work-related low back pain frequently leads to work absenteeism, lost productivity, work disability, early retirement and increased health care utilisation, which can negatively impact the quality of life and mental wellbeing of those affected [3–5]. A study in Australia found that 38% of participants working full time had to change their work status as a result of low back pain (LBP) [6]. Persistent cases of WRLBP increase the risk of negative consequences across many aspects of a person’s life [7]. These detrimental impacts may extend beyond the affected individual to their families, community, the healthcare system and labour market [5, 7, 8]. A review on indirect cost of WRLBP found that cost of work absenteeism was significant, ranging from 27.4% to 95% of total costs [9].

Many nations have established cause-based work injury compensation schemes to fund healthcare and treatment for injured and ill workers. Work-related low back pain is a very common condition leading to workers’ compensation claims [10, 11]. Within some compensation schemes such as those in Australia, Canada and the United States, General Practitioners (GPs) provide first line care for WRLBP and refer patients to specialists, allied health professionals, and diagnostic tests as needed for further management. GPs often also have additional administrative and regulatory responsibilities such as monitoring progress, undertaking work capacity assessments, reporting to compensation providers or insurers as well as providing advice to key stakeholders in the recovery and return to work process, and advocacy [12, 13]. Some of these additional roles and responsibilities are unique to compensation systems; for example, the provision of certificates of work capacity is a legislative requirement in Australian and Canadian workers’ compensation systems. These features of the compensation environment may impact on GP service utilisation. For example, a recent study comparing the frequency of GP service use in four Australian workers’ compensation schemes identified higher use in states that required GPs to provide medical certification of incapacity to work every 4 weeks, suggesting that system policy was influencing worker service utilisation [14].

Having an accepted workers’ compensation claim is associated with increased adherence to healthcare use [15], likely because workers’ compensation insurers fund healthcare costs, thus reducing a barrier to accessing care [16, 17]. Given that GP service use may be higher for people with WRLBP than for those whose back pain is not work-related, and that factors such as compensation system policy may influence service utilisation, it is important to understand the prevalence, frequency, duration and determinants of GP service use for WRLBP. Quantifying the burden that WRLBP places on GPs and health systems will be helpful in health system planning and resource allocation. This may in turn contribute to better outcomes in worker recovery and return to work, and a reduction in the individual, economic and societal burden resulting from WRLBP.

For the general population, GP service use is well studied and documented including studies of prevalence of GP service use for LBP management [18–22], timing of care [21] and determinants of GP service use [18]. However, there is a gap in knowledge when it comes to the utilisation of GP services among workers with WRLBP despite its huge burden and impact in the society. Thus, this systematic review aims to address these gaps in knowledge regarding the understanding of the prevalence, patterns (frequency and duration) and determinants of GP service use for WRLBP by synthesising the published, peer-reviewed research literature.

## Methods

The review protocol for this study was pre-registered in PROSPERO (CRD42023414494), the International Prospective Register for systematic reviews [23]. This review was conducted as per *Cochrane handbook for systematic review and interventions* guidelines [24] and was reported following Preferred Reporting Items for Systematic Reviews and Meta-Analysis (PRISMA) guidelines [25, 26].

## Data Source and Search Strategy

Four electronic databases (MEDLINE, Embase via Ovid, Scopus and Web of Science) were searched for peer-reviewed articles published in English with no restriction on the year of publication. Searches were conducted from inception to 21 April 2023. An expert librarian was consulted to search relevant literatures using optimised search strategy. The search terms were broadly categorised into three groups: Population (condition), Population (setting) and Outcome. The population (condition) included terms such as “low back pain” and “back pain”. The population (setting) consisted of terms such as “workplace”, “worker” and “employment”. The outcome terms included phrases such as “family practice”, “general practitioners” and “family physicians”. A detailed search strategy is attached as Appendix A. Boolean operators (OR, AND, and NOT) and controlled vocabulary terms (Emtree in Embase and MeSH terms in MEDLINE) were used where possible. Forward and backward citation searches were performed to avoid missing any relevant literature.

## Screening

All records from the different search databases were imported to EndNote reference management software [27] to remove duplicates. After removing duplicates, the relevant articles were uploaded to Covidence [28], an online review management software for screening and data extraction. Two reviewers independently conducted title and abstract screening to select studies for full-text review. Likewise, two independent reviewers conducted full-text screening using inclusion and exclusion criteria listed below to select studies for inclusion in the review. Any discrepancies in any stage of screening were resolved through discussion.

## Eligibility

For the purpose of this systematic review, GP service use was defined as any consultation with a physician, medical doctor or GP. Any reported LBP was considered eligible, including samples described as acute, subacute, or chronic/persistent. The eligibility criterion for LBP was met when more than 50% of study participants reported LBP. The LBP was considered attributed to work if 1) the study utilised workers’ compensation claims for analysis, specifically focusing on claims related to LBP, 2) study participants with accepted workers’ compensation claims for LBP were enrolled or 3) there was a reported connection between work and the LBP condition, suggesting LBP could be attributed to work. Only peer-reviewed original article published in English were included. Only observational studies involving participants aged 15 years older with WRLBP were considered for inclusion.

## Study Quality and Risk of Bias

We used a risk of bias assessment tool developed for prevalence studies [29]. The tool consists of 10 items with the first four items assessing external validity and the remaining six items assessing internal validity. Responses were recorded as “Yes” where risk of bias was considered to be low and “No” where it was considered to be high. The risk of bias was summarised as low risk (0 – 4 No), moderate risk (5 – 7 No) and high risk (8–10). For this review, some items were not applicable. For example, the likelihood of non-response bias was not applicable for the retrospective studies of entire populations. The risk of bias assessment was conducted by two reviewers separately and compared. Any disagreements between reviewers were resolved amicably through discussion. The risk of bias assessment is attached as Appendix B.

## Study Outcomes

The primary outcome of this study was the prevalence of GP service used by individuals with WRLBP, defined as the proportion of participants with WRLBP utilising at least one GP service measured over a time interval which can be indexed at an event (injury/claim) or a calendar date. The secondary outcomes were frequency, timing, duration and various determinants of GP services use among individuals with WRLBP.

## Data Extraction and Analysis

Two independent reviewers extracted data based on an agreed data extraction template attached as Appendix C and compared them for any inconsistency. All inconsistencies were resolved amicably via discussion. Recorded information consisted of author, publication year, country of study, study design, description of study population (dataset timeframe), sample size (total number of participants) and demographics (average age in years, male%). For primary outcome, prevalence period and prevalence of GP service use were recorded. In addition, information on frequency, timing, determinants and duration was extracted for secondary outcomes.

The heterogeneity of studies, particularly the different time periods used in the included studies and variations in study design, precluded quantitative synthesis. Narrative analysis was conducted by summarising extracted data in groups [30]. For the primary outcome, studies were grouped based on country of study considering the substantial differences in the healthcare system and workers’ compensation schemes across nations, which may affect prevalence. Furthermore, studies were grouped based on study design, source of study data and follow-up period to reflect the potential influence of these factors on observed prevalence of GP service use. For secondary outcomes such as frequency, duration and timing of care, the narrative analysis was conducted in two steps. Initially, studies reporting these secondary outcomes were grouped based on the follow-up period. Subsequently, these were grouped based on the statistical metrics such as mean with standard deviation or median with interquartile range or percentage of participants using GP services multiple times such as once, two to five or more than five, where applicable. In case of determinants of GP service use, the factors identified from the study were grouped into predisposing, enabling and need factors according to the definition of Anderson and Newman framework of healthcare service use [31]. This framework provides a structural approach for the complex interplay of predisposing factors (such as demographics, socioeconomic characteristics), enabling factors (such as income, insurance), need factors (such as perceived health status) and external factors (such as healthcare system, workers’ compensation system) that may determine the GP service use [31, 32].

## Results

### Selection of Included Studies

**Fig. 1:**
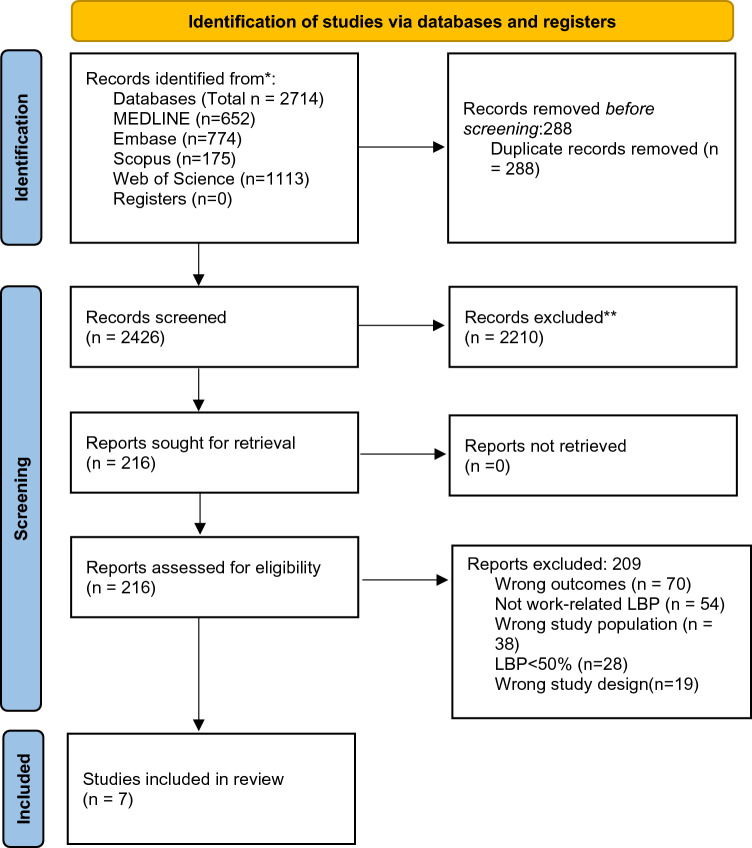
PRISMA flow diagram for study selection

The electronic database search across four different databases identified 2714 articles. After removing 288 duplicates, 2426 studies were included in title and abstract screening. Of these, 2210 studies were considered ineligible and excluded, leaving 216 studies for full-text screening. Of these, a further 209 were excluded from full-text screening following the inclusion and exclusion criteria, resulting in seven studies for the review. A flow diagram of selection of studies is shown in Fig. 1.

### Characteristics of Included Studies

Out of 7 included studies, as shown in Table 1, four were retrospective cohort studies [14, 33–35], two were prospective cohort studies [36, 37] and one was a cross-sectional study [17]. Three studies were conducted in the United States [17, 33, 35], two in Australia [14, 36] and two in Canada [34, 37]. Notably, the source of data differed across these studies. Three studies [14, 34, 37] used administrative workers’ compensation datasets, three studies [33, 35, 36] used medical records from GP clinics and one study [17] used a dataset provided by an employer. The number of participants ranged from 87 [33, 35] to 73,104 [14] with a total of 82,168 participants across all studies, of whom 53.5% were male. The age of the study participants ranged from 15 to 80, with average age ranging from 36.1 [33] to 40.6 years [14, 34]. Only one study [34] reported the WRLBP as acute and uncomplicated, while other studies did not specify the nature of LBP.

**Table 1:** Overview of study characteristics related to primary outcome

		Study population			Primary outcome	
Author, Year, Country,	Study design	Description of study population (dataset timeframe)	Sample size	Demographics: Average age, Gender	Prevalence period	Prevalence of General Practitioner service use (%)
Atlas 2004, the United States [33]	Retrospective case series	Workers’ compensation back pain patients at Massachusetts General Hospital owned four primary care centres (1996 to 1998)	87	36.1 years 68.9% male	Injury compensation claim date to 2 years later	39%
Blanchette 2016, Canada [34]	Retrospective Study	Workers who filed a lost-time claim with Workplace Safety and Insurance Board for uncomplicated back pain with a date of accident (01 January and 30 June 2015)	5520	39 years 61.9% male	Injury date to the date of the first healthcare provider accessed	85.3%
Collie 2022, Australia [14]	Retrospective Study	WRLBP claims with at least 1 day of recorded income support payment. (01 July 2010 to 30 June 2015)	73,104	40.6 (18–50) years 65.36% male	3 months before claim acceptance to 2 years after claim acceptance	70%
Cote 2005, the United States [17]	Cross – Sectional Study	Workers aged 18 + who received compensation claim for LBP and employed in American West Airlines, American Medical Responses, The Earth grains Co., Maricopa Country, and Marriott International, Inc. (01 July 1999 to 30 June 2002)	1104	37.95 years 49.2% male	Injury date until an interview date (between 4 and 16 weeks claim lodgement)	89.4%
Piterman 1987, Australia [36]	Prospective cohort study	Cohort of patients presented to Moorabbin clinic during the period 1 January – 31 December 1979, with low back injuries identified from the workers’ compensation certificates (unspecified to 1984)	119	37.2 years 85.71% male	Undefined – self reported as current treatment at five years follow-up after GP visit	11%
Rossignol 1996, Canada [37]	Prospective cohort study	Patients aged 15–65 years who were granted compensation by Quebec Workers’ Compensation Board for LBP resulting from a work-related injury with at least 1 day of disability from work between 1 January 1988 and 31 December 1988 (01 January 1988 to 31 December 1990)	2147	15 to 64 years 76.75% male	First day of compensated absence to 2 years later	99.3%
Wasiak 2008, the United States [35]	Retrospective case series	Individuals with workers compensation for low back pain claim filed with insurer and have at least 1 visit to primary care centre owned by Massachusetts General Hospital between 1 October 1995 and 30 September 1998	87	Not Reported	Injury date to claim closure date	25.3%

Relating to secondary outcomes (as depicted in Table 2), four studies [14, 35–37] reported the frequency of GP service use, three studies [17, 33, 34] reported determinants of GP service use, three studies [33, 34, 36] reported timing of initial GP service use after claim or injury and one study [14] reported the duration of GP service use. Studies varied substantially in time periods used to measure GP service use. Four studies [14, 33, 35, 37] recorded the GP service use over a two-year time period, although the initial date differed among these studies. Two studies focused on shorter durations: within 4 to 16 weeks after reporting WRLBP compensation claims [17] and at the first health care provider visit [34]. One study [36] reported GP service use at five years follow-up after their GP visit.

**Table 2:** Overview of study characteristics related to secondary outcomes

		Study population		Secondary outcomes		
Author, Year, Country,	Study design	Description of study population (dataset timeframe)	Sample size	Prevalence period	General Practitioner service frequency (mean(sd)), Timing of care (mean/median), duration (mean(sd))	Determinants
Atlas 2004, the United States [33]	Retrospective case series	Workers’ compensation back pain patients at Massachusetts General Hospital owned four primary care centres (1996 to 1998)	87	Injury compensation claim date to 2 years later	NR, Median = 47.5 days, Duration—NR	Gender, male – OR 1.4 (95% CI of 0.55 – 3.7) Any past psychiatry history – OR 1.6 (95% CI of 0.69 – 4.1), Any past low back pain history – OR 2.4 (95% CI of 0.97 – 6.1)
Blanchette 2016, Canada [34]	Retrospective Study	Workers who filed a lost-time claim with Workplace Safety and Insurance Board for uncomplicated back pain with a date of accident (01 January and 30 June 2015)	5520	Injury date to the date of the first healthcare provider accessed	NR, 2.1(3.9) days NR	Odds of first seeing a physiotherapist rather than a medical doctor (OR = 2.03)
Collie 2022, Australia [14]	Retrospective Study	WRLBP claims with at least 1 day of recorded income support payment. (1 July 2010 to 30 June 2015)	73,104	3 months before claim acceptance to 2 years after claim acceptance	9.6 (12.4) NR 29 (35) weeks	NR
Cote 2005, the United States [17]	Cross – Sectional Study	Workers aged 18 + who received compensation claim for LBP and employed in American West Airlines, American Medical Responses, The Earth grains Co., Maricopa Country, and Marriott International, Inc. (01 July 1999 to 30 June 2002)	1104	Injury date until an interview date (between 4 and 16 weeks claim lodgement)	NR, NR, NR	Transportation/moving sector is less likely than those with service jobs to consult a combination of medical physicians and others compared to physician alone Prior history of back pain more likely to use combination of services
Piterman 1987, Australia [36]	Prospective cohort study	Cohort of patients presented to Moorabbin clinic during the period 1 January – 31 December 1979, with low back injuries identified from the workers’ compensation certificates (unspecified to 1984)	119	Undefined – self reported as current treatment at five years follow-up after GP visit	2.6 ± 1.6 3.9 days NR	NR
Rossignol 1996, Canada [37]	Prospective cohort study	Patients aged 15–65 years who were granted compensation by Quebec Workers’ Compensation Board for LBP resulting from a work-related injury with at least 1 day of disability from work between 1 January 1988 and 31 December 1988 (01 January 1988 to 31 December 1990)	2147	First day of compensated absence to 2 years later	3.8 per patient, NR NR	NR
Wasiak 2008, the United States [35]	Retrospective case series	Individuals with workers compensation for low back pain claim filed with insurer and have at least 1 visit to primary care centre owned by Massachusetts General Hospital between 1 October 1995 and 30 September 1998	87	Injury date to claim closure date	3.82 ± 3.79, NR, NR	NR

*NR* not reported, *SD *standard deviation, *OR *odds ratio, *CI *confidence interval

### Risk of Bias

Out of the seven studies assessed for risk of bias, four [14, 33, 35, 37] were rated as low risk of bias, and three [17, 34, 36] were rated as moderate risk. *Atlas *et al. [33] and *Wasiak *et al. [35] were both rated as low risk of bias. These studies used same mode of data collection, used appropriate case definition, followed up participants for 2 years and used appropriate numerators and denominators. However, these studies have some concerns as the sampling frame was not a true or close representative of the target population which limits the generalisability of the findings. Among three studies rated as moderate risk of bias, two articles [17, 36] have some concerns over the likelihood of non-response and self-reporting of GP service use which may lead to the underestimation or overestimation of the outcome [38, 39].

### Primary Outcome—Prevalence of GP Service Use

Reported prevalence ranged from a low of 11% (at five year after visit to a GP clinic) [36] to a high of 99.3% (during two years after first day of compensated claim) [37], as presented in Table 1, which varied substantially by follow-up period. Prevalence was comparatively higher in studies with shorter follow-up period after filing a workers compensation ranging from 85% [34] to 89.4% [17]. The prevalence decreased with a longer time period, with prevalence at five years after attending a GP clinic reported as 11% [36]. Four studies [14, 33, 35, 37] with a two years follow-up period after injury or claim acceptance reported prevalence ranging from 25.3% [35] to 99.3% [37]. In these four studies, prevalence was notably higher in Canada and Australia with 99.3% [37] and 70% [14], respectively, compared to 25.3% [35] and 39% [33] in the United States. In contrast, there was no country-wise difference in studies [17, 34] with shorter follow-up periods. There was only one study [36] at five-year period; hence, it could not be compared.

Four studies [14, 17, 34, 37] using the administrative workers compensation dataset and employer provided dataset [17] had higher sample sizes (1104 to 73,104) and reported higher prevalence ranging from 70% [14] to 99.3% [37]. Conversely, three studies [33, 35, 36] using GP clinics data had lower sample sizes (87 to 119) and reported relatvively lower prevalence, ranging from a minimum of 11% [36] to a maximum of 39% [35].

### Secondary Outcomes

Four studies [14, 35–37] reported the frequency of GP service using number of visits (mean and standard deviation). The average number of GP service use during two years follow-up was higher in Australia 9.6 (12.4) [14] compared to Canada [37] and the United states [35]. Despite the reported higher prevalence of GP service use in Canada (99.30%) [37] compared to the United States (25.30%) [35], the mean number of GP services used was 3.80 (3.79) in both studies during the same follow-up period. Additionally, percentage of workers using GP service once (34.70% vs 36.36%), 2 to 5 times (47.40% vs 36.30%) and 6 or more (17.20% vs 27.27%) were similar [35, 37]. One study reported an average of 2.6 (1.6) visits during initial injury with unspecified time intervals [36], thus could not be compared.

Among the participants using GP services as the first health care provider, the timing to first GP service use was 2.1 days in average [34], while the study following up the GP services use for 2 years reported a median of 47.5 days [33]. Duration of GP service use varied from 29 weeks for claims with at least one day of recorded income support payment to 36.9 weeks for claims with at least 2 weeks of time loss [14].

### Determinants of GP Service Use

All three studies [17, 33, 34] conducted logistic regression and presented their findings as odds ratio. Predisposing factors that are positively associated with the increased use of GP services were younger age, and working in the transport sector. Younger workers were more likely to choose GP service as their first health care provider for WRLBP [34], and they were more likely to use combinations of health care services including GP services than older workers [17]. The odds of receiving GP service were less by 51% (95% CI 0.27–0.87) among males compared to their female counterparts. Workers in the transport sector were less likely to use GP services over combination of care (GP, physical therapist and chiropractor) than workers with service jobs [17].

Prior history of low back pain or similar injuries and more severe injuries was positively associated with increased use of GP services whereas prior GP visits were associated with decreased use of GP service use. Participants with prior history of low back pain were more likely (OR = 2.9; 95% CI 1.1–7.7) to use GP service [33] during 2 years follow-up period. In contrast, participants with prior similar injury were less likely to use GP services as the first health care provider compared to physical therapy service (OR = 1.71; 95% CI 1.25–2.33) and chiropractor service (OR = 1.60; 95% CI 1.34–1.90) [34]. Participants with a prior history of low back pain were more likely to use GP services in combination with other services such as physical therapy (OR = 1.49; 95% CI 1.08–2.04), chiropractor (OR = 1.29; 95% CI 0.70–2.38) or a combination of chiropractor and physical therapy (OR = 1.07; 95% CI 0.66–1.75) than GP services alone [17]. Injured workers with more severe injuries and greater functional limitations were more likely to receive care from a combination of chiropractors, medical physicians and physiotherapists than a GP alone, while workers with better health status were more likely to be treated by GPs only [17]. Prior GP visit was associated with decreased likelihood of GP service use (OR = 0.42, 95% CI 0.14–1.20) for subsequent LBP [33].

## Discussion

This systematic review identified a wide variation in the reported prevalence of GP service use, ranging from 11% at 5 years follow-up after an initial GP attendance to 99.3% during a 2-year follow-up after first day of compensated time off work. This high degree of variation reflects, in part, the diversity of methods used to measure prevalence. Studies with longer follow-up periods typically reported lower prevalence values. Despite all of our included studies involving workers with claims for compensation, we observed that the prevalence of GP service use during 2 years follow-up was higher in Australia and Canada compared to the United States, suggesting that policy variation between workers compensation systems in these countries may influence the use of GP services. This review also identified a higher frequency of GP service use in Australia, which may reflect the additional administrative requirements for GPs that have been reported in Australian workers’ compensation jurisdictions [14].

In Australia [20] and Canada [40], GP service for LBP among the general population is lower compared that among to injured workers with WRLBP. The comparatively high GP service use for WRLBP results in increased costs to workers’ compensation schemes and may add to the workload of already overburdened GP clinics, potentially lengthening wait times for appointments and reducing access to care for other patients. For the general population, the prevalence of GP service use is consistent across Australia, Canada and the United States [20, 40, 41]. This further suggests that the observed between-country variation in the current study may be due to variation in workers’ compensation scheme design. Both Canada and Australia have publicly operated and tightly regulated workers’ compensation systems [16] in which GPs have clearly defined roles in providing medical certification of fitness to work, and in which injured workers may choose the GP of their choice. In contrast, most systems in the United States adopt a more privatised approach to the delivery of workers’ compensation insurance, with less regulatory oversight by government (noting that approaches vary from state to state) [42]. In the United States, state law decides the number of physicians from whom the injured worker can seek treatment, whether the injured worker may visit a physician of their own choice or whether the injured worker must seek care from the physician contracted by the employer. In many states, the ability of workers to choose or switch GPs is restricted [43]. In contrast, in Australia and Canada, workers are able to select their own GP, and studies have observed that it is common for multiple different GPs to provide care during the course of a WRLBP compensation claim [44]. The observed between-country differences may also be attributed to the methodological variation in data sources used. The United States studies used data from medical records within specific GP clinics with small sample sizes, which may not accurately capture the broader population of workers who seek GP services outside of these clinics, or those covered by different insurance providers or employers. In contrast, studies from Canada and Australia used workers’ compensation administrative data with large sample sizes and broader data capture, potentially leading to higher reported prevalence of GP service use.

We used Anderson-Newman’s conceptual framework [31, 32] for exploring the factors influencing the GP service use for WRLBP. Despite the significant societal impact of WRLBP, there is a paucity of studies investigating the determinants of GP service use for WRLBP, with only three of the included studies reporting any determinants. Though it is well known that health system-level factors such as workers’ compensation scheme policy can impact GP service use, none of the included studies explored these enabling factors.

Instead, studies reported predisposing and need factors. Younger workers are more likely to choose GP as first healthcare provider and are more likely to use combinations of healthcare services including GP, compared to older workers. In contrast to our findings, prior evidence suggests that age has no impact on health-seeking behavior [45, 46], and one prior study reported that older people use more GP services for LBP [47]. Needs factors such as prior history of low back pain and greater severity of injuries are associated with increased service use. This is concordant with other studies that found a prior history of LBP is associated with higher healthcare use [46, 48]. A prior history of LBP is associated with higher risk of experiencing new episodes of LBP, thus increasing the overall healthcare service use [49]. More severe injuries are associated with higher use of GP services. This agrees with the studies [46–48, 50, 51] that reported nature and severity of back pain and pain intensity were greatly associated with the increased use of the healthcare services for LBP.

This review was conducted adhering to the best practice methods such as protocol registration before commencing literature search, screening by two independent reviewers, and data extraction by two independent reviewers. Included studies used workers’ compensation datasets and routinely collected medical records, which ensured that we gained data that represent the target population of workers with work-related low back pain. In addition, the use of information from registries reduces the recall bias as seen in self- reported surveys. The incorporation of study quality into our data synthesis focused on the interpretation of the most reliable estimates.

Though the review provides some insights on prevalence, frequency, duration and determinants, there are many gaps and inconsistencies in the evidence. Despite the acknowledged very important role that GPs play in the care of workers with WRLBP, there is a paucity of research on this specific cohort. Only seven studies from Australia, Canada and the United States were eligible. This may be because in many countries, workers’ compensation is integrated into the universal health coverage, and studies have not differentiated between work-related cases of LBP or other causes. Similarly, low- and middle-income countries may have additional challenges due to limited insurance or workers’ compensation coverage.The true prevalence of GP service use for WRLBP remains elusive mainly due to the variation in definitions of prevalence period, with no standard starting or ending points. The methodology in these studies was heterogeneous; for example, studies used different follow-up periods and data sources, and some included small sample sizes. The sample size varied from 87 to 73,104 which also partly accounted for the wide variation in outcome measure. Only one study specified the LBP type as acute and uncomplicated LBP which meant it was not possible to report the prevalence by type of LBP. In addition, although many studies focus on LBP, however, establishing LBP attributed to work is challenging, hindering the acquisition of information on GP services use for WRLBP. Included studies reported GP service use and accepted workers’ compensation, but lacked specificity regarding the purpose of GP visits, particularly in the data obtained from GP clinics, potentially leading to an underestimation or overestimation of GP services use for WRLBP. Additionally, this lack of clear delineation of GP service makes it unclear whether this increased GP service use is due to reporting and/or administrative requirements for workers’ compensation systems or increased severity of WRLBP under workers’ compensation claims or lack of access to other appropriate alternatives for the treatment. There is limited information on determinants of GP service use. Despite well-known influence of mandatory regulatory requirements by GPs and funding from workers’ compensation schemes for WRLBP on GP service use, these factors were not explored. Furthermore, there is no recent information as studies included data dating back from 1987 to 2015 and the prevalence of GP services may have changed over the past eight years and policies might have changed since then affecting the use of GP services. Finally, there is a lack of prospective data with majority of studies using retrospective data. Even those using prospective data have usually small sample size reducing the precision and generalisability of the findings.

Larger prospective longitudinal studies specific to these individuals with long-term healthcare needs would provide a more nuanced understanding of how individuals with WRLBP use GP services, how service use evolves over time and what the outcomes of these service use are compared to general population. More research using recent data needs to be conducted on facilitators and barriers such as perceived pain severity, complex workers compensation claim procedure, financial constraints, time constraints, self-reliance on self-care methods, perception, cultural aspects and belief about cause and effect.

## Conclusions

The prevalence of GP service use for WRLBP differs significantly across different studies ranging from 11% at five years follow-up after GP service use to 99.3% during two years follow-up. Despite the important role GPs play in WRLBP, evidence on prevalence was scarce, with only seven eligible studies identified. These studies contained very limited information on patterns and determinants of GP service use. Further research on GP service use for WRLBP is needed to understand the complete picture of GP service use among workers with WRLBP.

## Supplementary Information

Below is the link to the electronic supplementary material.

Supplementary file1 (PDF 144 KB)

## Data Availability

No datasets were generated or analysed during the current study

## References

[1] Driscoll T, Jacklyn G, Orchard J, Passmore E, Vos T, Freedman G, et al. The global burden of occupationally related low back pain: estimates from the Global Burden of Disease 2010 study. Ann Rheum Dis. 2014;73(6):975–981. 10.1136/annrheumdis-2013-204631 24665117

[2] Ferreira ML, de Luca K, Haile LM, Steinmetz JD, Culbreth GT, Cross M, et al. Global, regional, and national burden of low back pain, 1990–2020, its attributable risk factors, and projections to 2050: a systematic analysis of the Global Burden of Disease Study 2021. The Lancet Rheumatology. 2023;5(6):e316–e329. 10.1016/S2665-9913(23)00098-X PMC10234592 37273833

[3] Buchbinder R, Underwood M, Hartvigsen J, Maher CG. The Lancet Series call to action to reduce low value care for low back pain: an update. Pain. 2020;161(1):S57. 33090740 10.1097/j.pain.0000000000001869 PMC7434211

[4] Froud R, Patterson S, Eldridge S, Seale C, Pincus T, Rajendran D, et al. A systematic review and meta-synthesis of the impact of low back pain on people’s lives. BMC Musculoskelet Disord. 2014;15(1):1–14. 24559519 10.1186/1471-2474-15-50 PMC3932512

[5] Schofield DJ, Shrestha RN, Passey ME, Earnest A, Fletcher SL. Chronic disease and labour force participation among older Australians. Med J Aust. 2008;189(8):447–450. 10.5694/j.1326-5377.2008.tb02119.x 18928439

[6] Henschke N, Maher CG, Refshauge KM, Herbert RD, Cumming RG, Bleasel J, et al. Prognosis in patients with recent onset low back pain in Australian primary care: inception cohort study. BMJ. 2008:337. 10.1136/bmj.a171 PMC2483884 18614473

[7] Hartvigsen J, Hancock MJ, Kongsted A, Louw Q, Ferreira ML, Genevay S, et al. What low back pain is and why we need to pay attention. Lancet. 2018;391(10137):2356–2367. 10.1016/S0140-6736(18)30480-X. 10.1016/S0140-6736(18)30480-X 29573870

[8] Hoy D, Brooks P, Blyth F, Buchbinder R. The epidemiology of low back pain. Best Pract Res Clin Rheumatol. 2010;24(6):769–781. 10.1016/j.berh.2010.10.002 21665125

[9] Tymecka-Woszczerowicz A, Wrona W, Kowalski PM, Hermanowski T. Indirect costs of back pain – review. Polish Ann Med. 2015;22(2):143–148. 10.1016/j.poamed.2015.07.003.

[10] Di Donato M, Buchbinder R, Iles R, Gray S, Collie A. Low back pain in compensated Australian workers: a retrospective cohort study. medRxiv. 2020:2020.02. 24.20027540.

[11] Oakman J, Clune S, Stuckey R. Work-related musculoskeletal disorders in Australia. Canberra: Safe Work Australia; 2019.

[12] Kosny A, MacEachen E, Ferrier S, Chambers L. The role of health care providers in long term and complicated workers’ compensation claims. J Occup Rehabil. 2011;21:582–590. 10.1007/s10926-011-9307-3 21468735

[13] Mazza D, Brijnath B, Singh N, Kosny A, Ruseckaite R, Collie A. General practitioners and sickness certification for injury in Australia. BMC Fam Pract. 2015;16:1–9. 26275607 10.1186/s12875-015-0307-9 PMC4537596

[14] Collie A, Sheehan L, Di Donato M. Variation in general practice services provided to Australian workers with low back pain: a cross-jurisdictional comparative study. J Occup Rehabil. 2022;32(2):203–214. 10.1007/s10926-021-10013-8. 10.1007/s10926-021-10013-8 34800245

[15] Brezzi M, Luongo P. Regional disparities in access to health care: a multilevel analysis in selected OECD countries. 2016.

[16] Collie A, Lane T. Australian workers’ compensation systems. Understanding the Australian Health Care System Chatswood. Elsevier Australia: NSW, 2019, p. 208–222.

[17] Cote P, Baldwin ML, Johnson WG. Early patterns of care for occupational back pain. Spine (Philadelphia, Pa 1976). 2005;30(5):581–587. 10.1097/01.brs.0000154613.17511.dd. 10.1097/01.brs.0000154613.17511.dd 15738794

[18] Beyera GK, O’Brien J, Campbell S. Health-care utilisation for low back pain: a systematic review and meta-analysis of population-based observational studies. Rheumatol Int. 2019;39(10):1663–1679. 10.1007/s00296-019-04430-5 31463608

[19] Fullen B, Morlion B, Linton SJ, Roomes D, van Griensven J, Abraham L, et al. Management of chronic low back pain and the impact on patients’ personal and professional lives: Results from an international patient survey. Pain Pract. 2022;22(4):463–477. 10.1111/papr.13103 PMC9306505 35156770

[20] Haas R, Gorelik A, Busija L, O’Connor D, Pearce C, Mazza D, et al. Prevalence and characteristics of musculoskeletal complaints in primary care: an analysis from the population level and analysis reporting (POLAR) database. BMC Primary Care. 2023;24(1):40. 36739379 10.1186/s12875-023-01976-z PMC9898983

[21] Kamal KC, Alexandru DO, Kamal D, Maria DT, Kamal AM, Radu M, et al. Managing low back pain in primary care. Curr Health Sci J. 2020;46(4):396. 33717515 10.12865/CHSJ.46.04.11 PMC7948016

[22] Michaleff ZA, Harrison C, Britt H, Lin C-WC, Maher CG. Ten-year survey reveals differences in GP management of neck and back pain. European Spine J. 2012;21:1283–1289. 10.1007/s00586-011-2135-5 PMC3389113 22228573

[23] Maharjan P, Griffiths D, Collie A: Use of General Practitioner services for work-related low back pain: a systematic review and meta-analysis. https://www.crd.york.ac.uk/prospero/display_record.php?ID=CRD42023414494 (2023). Accessed. 10.1007/s10926-024-10187-x PMC11839839 38652423

[24] Higgins JP, Thomas J, Chandler J, Cumpston M, Li T, Page MJ, et al. Cochrane handbook for systematic reviews of interventions. Wiley; 2019. 10.1002/14651858.ED000142 PMC10284251 31643080

[25] Page MJ, McKenzie JE, Bossuyt PM, Boutron I, Hoffmann TC, Mulrow CD, et al. The PRISMA 2020 statement: an updated guideline for reporting systematic reviews. Int J Surg. 2021;88: 105906. 33789826 10.1016/j.ijsu.2021.105906

[26] Page MJ, Moher D, Bossuyt PM, Boutron I, Hoffmann TC, Mulrow CD, et al. PRISMA 2020 explanation and elaboration: updated guidance and exemplars for reporting systematic reviews. BMJ. 2021;372: n160. 10.1136/bmj.n160. 33781993 10.1136/bmj.n160 PMC8005925

[27] Gotschall T. EndNote 20 desktop version. JMLA. 2021;109(3):520. 34629985 10.5195/jmla.2021.1260 PMC8485940

[28] Babineau J. Product review: Covidence (systematic review software). J Canad Health Lib Assoc/Journal de l’Association des bibliothèques de la santé du Canada. 2014;35(2):68–71.

[29] Hoy D, Brooks P, Woolf A, Blyth F, March L, Bain C, et al. Assessing risk of bias in prevalence studies: modification of an existing tool and evidence of interrater agreement. J Clin Epidemiol. 2012;65(9):934–939. 10.1016/j.jclinepi.2011.11.014 22742910

[30] Cumpston MS, Brennan SE, Ryan R, McKenzie JE. Synthesis methods other than meta-analysis were commonly used but seldom specified: survey of systematic reviews. J Clin Epidemiol. 2023. 10.1016/j.jclinepi.2023.02.003 36758885

[31] Andersen R, Newman JF. Andersen and Newman framework of health services utilization. J Health Social Behav. 1995;36(March):1–10.

[32] Andersen R, Newman JF. Societal and individual determinants of medical care utilization in the United States. The Milbank Mem Fund Quart Health Soc 1973:95–124. 4198894

[33] Atlas SJ, Wasiak R, van den Ancker M, Webster B, Pransky G. Primary care involvement and outcomes of care in patients with a workers’ compensation claim for back pain. Spine. 2004;29(9):1041–1048. 10.1097/00007632-200405010-00017 15105679

[34] Blanchette M-A, Rivard M, Dionne CE, Hogg-Johnson S, Steenstra I. Workers’ characteristics associated with the type of healthcare provider first seen for occupational back pain. BMC Musculoskelet Disord. 2016;17(1):428. 27756318 10.1186/s12891-016-1298-y PMC5069865

[35] Wasiak R, Pransky GS, Atlas SJ. Who’s in charge? Challenges in evaluating quality of primary care treatment for low back pain. J Eval Clin Pract. 2008;14(6):961–968. 10.1111/j.1365-2753.2007.00890.x. 10.1111/j.1365-2753.2007.00890.x 18373572

[36] Piterman L, Dunt D. Occupational lower-back injuries in a primary medical care setting: a five-year follow-up study. Med J Aust. 1987;147(6):276–279. 10.5694/j.1326-5377.1987.tb133454.x 2957571

[37] Rossignol M, Abenhaim L, Bonvalot Y, Gobeille D, Shrier I. Should the gap be filled between guidelines and actual practice for management of low back pain in primary care? The Quebec experience Spine. 1996;21(24):2893–2899. 10.1097/00007632-199612150-00021 9112714

[38] Bhandari A, Wagner T. Self-reported utilization of health care services: improving measurement and accuracy. Med Care Res Rev. 2006;63(2):217–235. 10.1177/1077558705285298 16595412

[39] Brusco NK, Watts JJ. Empirical evidence of recall bias for primary health care visits. BMC Health Serv Res. 2015;15(1):1–8. 26373712 10.1186/s12913-015-1039-1 PMC4572632

[40] Wong JJ, Côté P, Tricco AC, Watson T, Rosella LC. Assessing the validity of health administrative data compared to population health survey data for the measurement of low back pain. Pain. 2021;162(1):219. 32910631 10.1097/j.pain.0000000000002003 PMC7737881

[41] Licciardone JC. The epidemiology and medical management of low back pain during ambulatory medical care visits in the United States. Osteopathic Med Primary Care. 2008;2(1):1–17. 10.1186/1750-4732-2-11 PMC2631527 19025636

[42] Ridic G, Gleason S, Ridic O. Comparisons of health care systems in the United States, Germany and Canada. Materia socio-medica. 2012;24(2):112. 23678317 10.5455/msm.2012.24.112-120 PMC3633404

[43] Shraim M, Cifuentes M, Willetts JL, Marucci-Wellman HR, Pransky G. Length of disability and medical costs in low back pain. J Occup Environ Med. 2015;57(12):1275–1283. 10.1097/JOM.0000000000000593 26492383

[44] Sheehan LR, Di Donato M, Gray SE, Lane TJ, Van Vreden C, Collie A. The association between continuity of care with a primary care physician and duration of work disability for low back pain: a retrospective cohort study. J Occup Environ Med. 2022;64(10):e606–e612. 10.1097/JOM.0000000000002643 35901194

[45] Adamson J, Hunt K, Nazareth I. The influence of socio-demographic characteristics on consultation for back pain—a review of the literature. Fam Pract. 2011;28(2):163–171. 10.1093/fampra/cmq085 PMC3062780 20974654

[46] Ferreira ML, Machado G, Latimer J, Maher C, Ferreira PH, Smeets RJ. Factors defining care-seeking in low back pain–a meta-analysis of population based surveys. Eur J Pain. 2010;14(7):747.e1–747.e7. 10.1016/j.ejpain.2009.11.005 20036168

[47] Walker BF, Muller R, Grant WD. Low back pain in Australian adults health provider utilization and care seeking. J Manipul Physiol Therap. 2004;27(5):327–335. 10.1016/j.jmpt.2004.04.006 15195040

[48] Molano S, Burdorf A, Elders L. Factors associated with medical care-seeking due to low-back pain in scaffolders. Am J Ind Med. 2001;40(3):275–281. 10.1002/ajim.1099 11598974

[49] Papageorgiou AC, Croft PR, Thomas E, Ferry S, Jayson M IV, Silman AJ. Influence of previous pain experience on the episode incidence of low back pain: results from the South Manchester Back Pain Study. PAIN®. 1996;66(2–3):181–185. 10.1016/0304-3959(96)03022-9 8880839

[50] Mannion AF, Wieser S, Elfering A. Association between beliefs and care-seeking behavior for low back pain. Spine. 2013;38(12):1016–1025. 10.1097/BRS.0b013e31828473b5 23459138

[51] Woodhouse A, Pape K, Romundstad PR, Vasseljen O. Health care contact following a new incident neck or low back pain episode in the general population; the HUNT study. BMC Health Serv Res. 2016;16(1):1–10. 26955969 10.1186/s12913-016-1326-5 PMC4782331

