# Supplementary material for: Use of General Practitioner Services Among Workers with Work-Related Low Back Pain: A Systematic Review
Source: J Occup Rehabil. 2024 Apr 23;35(1):4–16. doi: 10.1007/s10926-024-10187-x (PMC11839839; doi:10.1007/s10926-024-10187-x)
Supplement: Supplementary file 1 — Supplementary file1 (PDF 144 KB) [file 10926_2024_10187_MOESM1_ESM.pdf]

Appendix A: Search strategy: Ovid Medline

#	Query
1	Primary Health Care/
2	Family Practice/
3	General Practitioners/
4	Physicians, Family/
5	(GP or general practitioner* or family doctor* or family physician* or general practice or primary health or primary care or primary medical care or family practitioner).mp.
6	Low Back Pain/
7	Back Pain/
8	((pain* or ache* or aching) adj2 (low* back or lowback)).mp.
9	((Musculoskeletal or musculo-skeletal) adj2 pain*).mp.
10	Occupational Diseases/
11	Occupational Injuries/
12	Accidents, Occupational/
13	Workplace/
14	Work/
15	Workers' Compensation/
16	(work or job or workplace or work-place or occupation* or employment).mp.
17	((work* or job or employ*) adj2 (compensat* or claim* or cover* or disabilit* or liability insurance)).mp.
18	worker*.mp.
19	((sick* or ill* or medical) adj2 (absen* or leave)).mp.
20	1 or 2 or 3 or 4 or 5
21	6 or 7 or 8 or 9
22	10 or 11 or 12 or 13 or 14 or 15 or 16 or 17 or 18 or 19
23	20 and 21 and 22
24	limit 23 to english language
25	limit 24 to (case reports or clinical conference or comment or editorial or letter or news or newspaper article)
26	24 not 25

Appendix B: Risk of bias assessment

Study ID	Q1. Was the study's target population a close representation of the national population in relation to relevant variables, e.g. age, sex, occupation?	Q2 Was the sampling frame a true or close representative of the target population?	Q3. Was some form of random selection used to select the sample OR was a census undertaken?	Q4 Was the likelihood of non-response bias minimal?	Q5 Were data collected directly from the subjects (as opposed to a proxy)?	Q6 Was an acceptable case definition used in the study?	Q7 Was the study instrument that measured the parameter of interest shown to have validity and reliability?	Q8 Was the same mode of data collection used for all subjects?	Q9 Was the length of the shortest prevalence period for the parameter of interest appropriate?	Q10 Were the numerators and denominators for the parameter of interest appropriate?	Number of No	Remarks
Atlas 2004	No	No	Not applicable	Not applicable	Yes	Yes	Not applicable	Yes	Yes	Yes	2	Low risk of bias
Blanchette 2016	No	Yes	Not applicable	Not applicable	Yes	No	Not applicable	Yes	No	No	4	Moderate risk of bias
Collie 2022	No	Yes	Not applicable	Not applicable	Yes	Yes	Not applicable	Yes	Yes	Yes	2	Low risk of bias
Cote 2005	No	No	Not applicable	No	Yes	Yes	No	No	Yes	Yes	5	Moderate risk of bias
Piterman 1987	No	No	Not applicable	No	Yes	Yes	No	Yes	No	No	6	Moderate risk of bias
Rossignol 1996	No	Yes	Yes	Not applicable	Yes	Yes	Not applicable	Yes	Yes	No	2	Low risk of bias
Wasiak 2008	No	No	Not applicable	Not applicable	Yes	Yes	Not applicable	Yes	Yes	Yes	2	Low risk of bias

Appendix C: Data Extraction Template

Author, Year	Country of Study	Funding source	Conflict of interest	Study Design	Data collection method	Prevalence Period	Sample size	Age (mean(sd))	Age in Range	Male %	No of participants using at least 1 GP service	Prevalence %	Frequency	Timing of care (mean(sd))	Duration (weeks)	Determinants (OR)
